# Dicyclo­hexyl[*N*-(3-meth­oxy-2-oxido­benzyl­idene)valinato-κ^3^ *O*,*N*,*O*′]tin(IV)

**DOI:** 10.1107/S1600536809008393

**Published:** 2009-03-14

**Authors:** Hong-Jun Yang, Yan-Qiu Dang

**Affiliations:** a Research Center of Eco-Environmental Sciences Yellow River Delta, Binzhou University, Binzhou 256600, People’s Republic of China; b Department of Chemistry & Chemical Engineering, Binzhou University, Binzhou 256600, People’s Republic of China

## Abstract

In the title compound, [Sn(C_6_H_11_)_2_(C_13_H_15_NO_4_)], the Sn atom is five-coordinate and adopts a distorted trigonal-bipyramidal SnNC_2_O_2_ geometry with the O atoms in axial positions. The metal atom forms five- and six-membered chelate rings with the *O*,*N*,*O*′-tridentate ligand. The two cyclo­hexyl groups bound to the Sn atom adopt chair conformations, with the Sn—C bonds in equatorial positions and a mean Sn—C distance of 2.138 (3) Å.

## Related literature

For background to the chemistry of organotin Schiff base complexes, see: Beltran *et al.* (2003 ▶); Basu Baul *et al.* (2007 ▶); Dakternieks *et al.* (1998 ▶); Tian *et al.* (2005 ▶, 2006 ▶, 2007 ▶, 2009 ▶). For related structures, see: Li & Tian (2008 ▶); Tian *et al.* (2004 ▶, 2007 ▶).
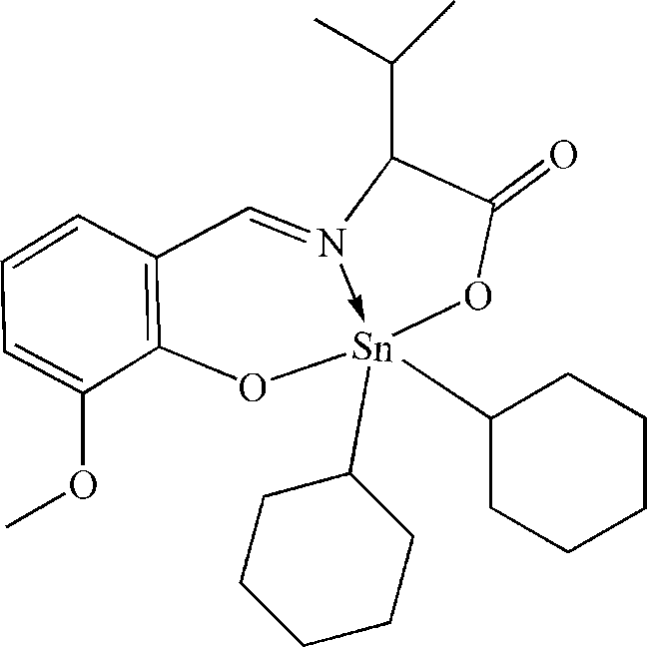


## Experimental

### 

#### Crystal data

[Sn(C_6_H_11_)_2_(C_13_H_15_NO_4_)]
*M* *_r_* = 534.25
Monoclinic, 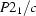

*a* = 9.5354 (5) Å
*b* = 10.0011 (6) Å
*c* = 25.7662 (15) Å
β = 94.345 (1)°
*V* = 2450.1 (2) Å^3^
*Z* = 4
Mo *K*α radiation
μ = 1.07 mm^−1^
*T* = 295 K
0.14 × 0.10 × 0.10 mm

#### Data collection

Bruker SMART APEX area-detector diffractometer
Absorption correction: multi-scan (*SADABS*; Bruker, 2002 ▶) *T*_min_ = 0.864, *T*_max_ = 0.900
18670 measured reflections
4808 independent reflections
3858 reflections with *I* > 2σ(*I*)
*R*_int_ = 0.025

#### Refinement

*R*[*F* ^2^ > 2σ(*F* ^2^)] = 0.033
*wR*(*F* ^2^) = 0.077
*S* = 1.04
4808 reflections
280 parameters
H-atom parameters constrained
Δρ_max_ = 0.57 e Å^−3^
Δρ_min_ = −0.34 e Å^−3^

### 

Data collection: *SMART* (Bruker, 2002 ▶); cell refinement: *SAINT* (Bruker, 2002 ▶); data reduction: *SAINT*; program(s) used to solve structure: *SHELXS97* (Sheldrick, 2008 ▶); program(s) used to refine structure: *SHELXL97* (Sheldrick, 2008 ▶); molecular graphics: *ORTEP-3 for Windows* (Farrugia, 1997 ▶); software used to prepare material for publication: *SHELXL97*.

## Supplementary Material

Crystal structure: contains datablocks global, I. DOI: 10.1107/S1600536809008393/sj2591sup1.cif

Structure factors: contains datablocks I. DOI: 10.1107/S1600536809008393/sj2591Isup2.hkl

Additional supplementary materials:  crystallographic information; 3D view; checkCIF report

## Figures and Tables

**Table:** 

Sn1—O3	2.1085 (19)
Sn1—C7	2.135 (3)
Sn1—C1	2.142 (3)
Sn1—O1	2.157 (2)
Sn1—N1	2.172 (2)

**Table:** 

O3—Sn1—C7	95.63 (10)
O3—Sn1—C1	95.31 (9)
C7—Sn1—C1	121.20 (12)
O3—Sn1—O1	157.22 (8)
C7—Sn1—O1	92.06 (10)
C1—Sn1—O1	98.96 (10)
O3—Sn1—N1	82.79 (8)
C7—Sn1—N1	119.27 (11)
C1—Sn1—N1	119.38 (10)
O1—Sn1—N1	74.75 (8)

**Fig. 1.**
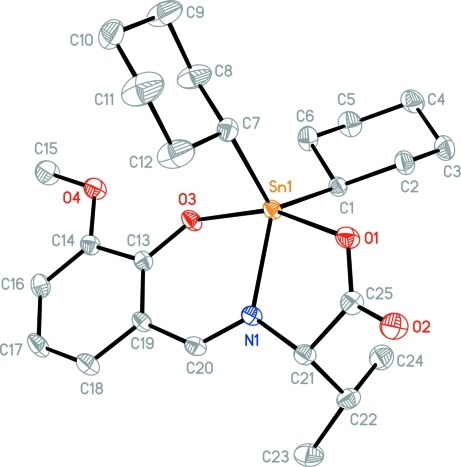
The structure of (I) with displacement ellipsoids are drawn at the 30% probability level. H atoms have been omitted for clarity.

## supplementary crystallographic information

### Comment

Diorganotin complexes with Schiff bases derived from α-amino acids continue to received attention because of their structural variety and biological activities (Beltran *et al.*, 2003; Basu Baul *et al.*, 2007; Dakternieks *et al.*, 1998; Tian *et al.*, 2005, 2006, 2007, 2009). The structures of three dicyclohexyltin complexes with the Schiff base ligand, [*N*-(5-chloro-2-oxidophenylmethylene)isoleucinato]dicyclohexyltin(IV) (Tian *et al.*, 2004), [*N*-(3,5-dibromo-2-oxidophenylmethylene)alaninato]dicyclohexyltin(IV) (Tian *et al.*, 2007) and [*N*-(5-chloro-2-oxidophenylmethylene)valinato]dicyclohexyltin(IV) (Li & Tian, 2008), have been reported. As a continuation of these studies, the structure of the title compound, (I), is reported here.

The coordination geometry of the tin atom in (I) is distorted trigonal bipyramidal with two cyclohexyl groups and the imino N1 atom occupying the equatorial positions and the axial positions being occupied by a unidentate carboxylate O1 atom and phenolate O3 atom (Fig. 1). The tin atom is 0.049 (2) Å out of the NC2 trigonal plane in the direction of the O3 atom. The bond length Sn1—O1 (2.157 (2) Å) was longer than that of Sn1—O3 (2.1085 (19) Å). The bond angle O1—Sn1—O3 was 157.22 (8)°, slightly larger than those found in [*N*-(5-chloro-2-oxidophenylmethylene)isoleucinato]dicyclohexyltin(IV) [153.84 (12)°] (Tian *et al.*, 2004), [*N*-(3,5-dibromo-2-oxidophenylmethylene)alaninato]dicyclohexyltin(IV) [154.9 (1)°] (Tian *et al.*, 2007) and [*N*-(5-chloro-2-oxidophenylmethylene)valinato]dicyclohexyltin(IV) [155.75 (12)°] (Li & Tian, 2008). The two cyclohexyl groups bound to the tin atom adopt chair conformations with the Sn—C bonds in equatorial positions with a mean distance of 2.138 (3) Å. The monodentate mode of coordination of the carboxylate is reflected in the disparate C23—O1 and C23—O2 bond lengths of 1.289 (4) and 1.217 (4) Å, respectively.

### Experimental

The title compound was prepared by the reaction of dicyclohexyltin dichloride (0.71 g, 2 mmol) with potassium *N*-(3-methoxy-2-hydroxyphenylmethylene)valinate (0.54 g, 2 mmol) in the presence of Et_3_N (0.20 g, 2 mmol) in methanol (30 ml). The reaction mixture was refluxed for 2 h and filtered. The yellow solid, (I),obtained by removal of solvent under reduced pressure, was recrystallized from methanol. Crystals of (I) suitable for X-ray measurements were obtained from dichloromethane-hexane (1:1, V/V) by slow evaporation at room temperature (yield 70%, m.p. 477–478 K).

### Refinement

H atoms were placed at calculated positions (C—H = 0.93–0.98 Å) and refined as riding with *U*_iso_(H) = 1.2Ueq(C) or 1.5Ueq(methyl C).

### Crystal data

**Table:**

[Sn(C_6_H_11_)_2_(C_13_H_15_NO_4_)]	*F*(000) = 1104
*M**_r_* = 534.25	*D*_x_ = 1.448 Mg m^−^^3^
Monoclinic, *P*2_1_/*c*	Mo *K*α radiation, λ = 0.71073 Å
Hall symbol: -P 2ybc	Cell parameters from 6982 reflections
*a* = 9.5354 (5) Å	θ = 2.3–27.2°
*b* = 10.0011 (6) Å	µ = 1.07 mm^−^^1^
*c* = 25.7662 (15) Å	*T* = 295 K
β = 94.345 (1)°	Block, yellow
*V* = 2450.1 (2) Å^3^	0.14 × 0.10 × 0.10 mm
*Z* = 4	

### Data collection

**Table:**

Bruker SMART APEX area-detector diffractometer	4808 independent reflections
Radiation source: fine-focus sealed tube	3858 reflections with *I* > 2σ(*I*)
graphite	*R*_int_ = 0.025
φ and ω scans	θ_max_ = 26.0°, θ_min_ = 1.6°
Absorption correction: multi-scan (*SADABS*; Bruker, 2002)	*h* = −11→11
*T*_min_ = 0.864, *T*_max_ = 0.900	*k* = −12→12
18670 measured reflections	*l* = −31→31

### Refinement

**Table:**

Refinement on *F*^2^	Primary atom site location: structure-invariant direct methods
Least-squares matrix: full	Secondary atom site location: difference Fourier map
*R*[*F*^2^ > 2σ(*F*^2^)] = 0.033	Hydrogen site location: inferred from neighbouring sites
*wR*(*F*^2^) = 0.077	H-atom parameters constrained
*S* = 1.04	*w* = 1/[σ^2^(*F*_o_^2^) + (0.0302*P*)^2^ + 2.2424*P*] where *P* = (*F*_o_^2^ + 2*F*_c_^2^)/3
4808 reflections	(Δ/σ)_max_ = 0.001
280 parameters	Δρ_max_ = 0.57 e Å^−^^3^
0 restraints	Δρ_min_ = −0.34 e Å^−^^3^

### Special details

**Table:**

Geometry. All e.s.d.'s (except the e.s.d. in the dihedral angle between two l.s. planes) are estimated using the full covariance matrix. The cell e.s.d.'s are taken into account individually in the estimation of e.s.d.'s in distances, angles and torsion angles; correlations between e.s.d.'s in cell parameters are only used when they are defined by crystal symmetry. An approximate (isotropic) treatment of cell e.s.d.'s is used for estimating e.s.d.'s involving l.s. planes.
Refinement. Refinement of *F*^2^ against ALL reflections. The weighted *R*-factor *wR* and goodness of fit *S* are based on *F*^2^, conventional *R*-factors *R* are based on *F*, with *F* set to zero for negative *F*^2^. The threshold expression of *F*^2^ > σ(*F*^2^) is used only for calculating *R*-factors(gt) *etc*. and is not relevant to the choice of reflections for refinement. *R*-factors based on *F*^2^ are statistically about twice as large as those based on *F*, and *R*- factors based on ALL data will be even larger.

### Fractional atomic coordinates and isotropic or equivalent isotropic displacement parameters (Å^2^)

**Table:**

	*x*	*y*	*z*	*U*_iso_*/*U*_eq_	
Sn1	0.13269 (2)	−0.000285 (19)	0.145122 (7)	0.03534 (8)	
N1	0.0746 (2)	0.2013 (2)	0.16634 (9)	0.0356 (5)	
O1	0.0070 (2)	−0.0239 (2)	0.21069 (8)	0.0469 (5)	
O3	0.2487 (2)	0.1017 (2)	0.09093 (8)	0.0438 (5)	
O4	0.4527 (3)	0.1480 (2)	0.03098 (9)	0.0557 (6)	
O2	−0.1148 (3)	0.0751 (2)	0.26995 (8)	0.0583 (6)	
C19	0.2495 (3)	0.3275 (3)	0.12149 (11)	0.0380 (7)	
C13	0.2988 (3)	0.2235 (3)	0.09125 (11)	0.0369 (6)	
C25	−0.0502 (3)	0.0784 (3)	0.23099 (12)	0.0431 (7)	
C14	0.4080 (3)	0.2540 (3)	0.05838 (12)	0.0421 (7)	
C7	0.3103 (3)	−0.0932 (3)	0.18570 (12)	0.0437 (7)	
H7	0.2734	−0.1674	0.2054	0.052*	
C21	−0.0371 (3)	0.2112 (3)	0.20312 (11)	0.0405 (7)	
H21	−0.0090	0.2795	0.2292	0.049*	
C20	0.1414 (3)	0.3096 (3)	0.15649 (11)	0.0407 (7)	
H20	0.1160	0.3856	0.1744	0.049*	
C1	0.0021 (3)	−0.1073 (3)	0.08792 (11)	0.0389 (7)	
H1	−0.0630	−0.0432	0.0703	0.047*	
C16	0.4606 (4)	0.3813 (3)	0.05633 (13)	0.0515 (8)	
H16	0.5319	0.3996	0.0347	0.062*	
C18	0.3057 (4)	0.4573 (3)	0.11829 (13)	0.0513 (8)	
H18	0.2718	0.5257	0.1384	0.062*	
C2	−0.0854 (4)	−0.2153 (3)	0.11246 (13)	0.0536 (8)	
H2A	−0.0232	−0.2777	0.1316	0.064*	
H2B	−0.1453	−0.1742	0.1368	0.064*	
C24	−0.2320 (4)	0.1559 (4)	0.13339 (15)	0.0630 (10)	
H24A	−0.2323	0.0666	0.1471	0.095*	
H24B	−0.1710	0.1600	0.1055	0.095*	
H24C	−0.3257	0.1803	0.1206	0.095*	
C6	0.0909 (4)	−0.1689 (4)	0.04705 (13)	0.0544 (9)	
H6A	0.1404	−0.0984	0.0301	0.065*	
H6B	0.1605	−0.2282	0.0641	0.065*	
C17	0.4089 (4)	0.4834 (3)	0.08615 (15)	0.0585 (9)	
H17	0.4450	0.5695	0.0841	0.070*	
C5	0.0011 (4)	−0.2466 (4)	0.00641 (13)	0.0648 (10)	
H5A	−0.0607	−0.1852	−0.0135	0.078*	
H5B	0.0614	−0.2891	−0.0174	0.078*	
C15	0.5593 (5)	0.1721 (4)	−0.00362 (16)	0.0716 (12)	
H15A	0.5818	0.0900	−0.0204	0.107*	
H15B	0.6418	0.2061	0.0156	0.107*	
H15C	0.5261	0.2363	−0.0294	0.107*	
C10	0.6078 (4)	−0.1408 (4)	0.21768 (18)	0.0750 (12)	
H10A	0.6546	−0.0709	0.1994	0.090*	
H10B	0.6794	−0.1924	0.2376	0.090*	
C22	−0.1800 (3)	0.2520 (3)	0.17606 (13)	0.0486 (8)	
H22	−0.2483	0.2490	0.2026	0.058*	
C23	−0.1805 (4)	0.3944 (4)	0.15532 (16)	0.0703 (11)	
H23A	−0.2718	0.4149	0.1390	0.105*	
H23B	−0.1113	0.4028	0.1303	0.105*	
H23C	−0.1589	0.4553	0.1836	0.105*	
C3	−0.1757 (4)	−0.2901 (4)	0.07076 (15)	0.0634 (10)	
H3A	−0.2281	−0.3597	0.0870	0.076*	
H3B	−0.2427	−0.2287	0.0534	0.076*	
C8	0.4100 (4)	−0.1546 (4)	0.14941 (15)	0.0752 (12)	
H8A	0.4481	−0.0847	0.1285	0.090*	
H8B	0.3586	−0.2165	0.1261	0.090*	
C11	0.5120 (5)	−0.0791 (6)	0.2538 (2)	0.1082 (19)	
H11A	0.5648	−0.0166	0.2765	0.130*	
H11B	0.4752	−0.1482	0.2754	0.130*	
C4	−0.0867 (4)	−0.3524 (4)	0.03086 (15)	0.0636 (10)	
H4A	−0.0253	−0.4196	0.0475	0.076*	
H4B	−0.1472	−0.3959	0.0040	0.076*	
C9	0.5308 (5)	−0.2287 (5)	0.17957 (18)	0.0885 (15)	
H9A	0.4934	−0.3044	0.1976	0.106*	
H9B	0.5951	−0.2626	0.1553	0.106*	
C12	0.3897 (5)	−0.0058 (5)	0.22460 (19)	0.0894 (16)	
H12A	0.3260	0.0266	0.2494	0.107*	
H12B	0.4257	0.0711	0.2069	0.107*	

### Atomic displacement parameters (Å^2^)

**Table:**

	*U*^11^	*U*^22^	*U*^33^	*U*^12^	*U*^13^	*U*^23^
Sn1	0.04106 (12)	0.03172 (11)	0.03376 (12)	−0.00233 (9)	0.00626 (8)	0.00054 (9)
N1	0.0449 (14)	0.0325 (13)	0.0302 (12)	−0.0001 (11)	0.0070 (10)	0.0002 (10)
O1	0.0621 (14)	0.0376 (12)	0.0432 (12)	0.0028 (10)	0.0181 (11)	0.0065 (9)
O3	0.0596 (13)	0.0322 (11)	0.0419 (11)	−0.0084 (10)	0.0176 (10)	−0.0006 (9)
O4	0.0687 (15)	0.0456 (13)	0.0569 (14)	−0.0032 (11)	0.0323 (12)	0.0013 (11)
O2	0.0775 (16)	0.0562 (15)	0.0449 (13)	0.0047 (13)	0.0294 (12)	0.0076 (11)
C19	0.0478 (17)	0.0313 (15)	0.0347 (15)	−0.0052 (13)	0.0023 (13)	0.0023 (12)
C13	0.0415 (16)	0.0360 (16)	0.0328 (15)	−0.0029 (13)	0.0002 (12)	0.0041 (12)
C25	0.0475 (17)	0.0476 (19)	0.0344 (16)	0.0008 (15)	0.0036 (13)	0.0056 (14)
C14	0.0479 (18)	0.0403 (17)	0.0389 (16)	−0.0024 (14)	0.0079 (14)	0.0069 (13)
C7	0.0422 (17)	0.0491 (18)	0.0397 (17)	−0.0025 (14)	0.0020 (13)	0.0116 (14)
C21	0.0497 (18)	0.0377 (16)	0.0352 (16)	0.0014 (14)	0.0111 (13)	−0.0014 (13)
C20	0.0511 (18)	0.0322 (16)	0.0390 (16)	0.0006 (13)	0.0044 (14)	−0.0040 (13)
C1	0.0394 (16)	0.0370 (16)	0.0403 (16)	−0.0031 (13)	0.0039 (13)	−0.0018 (13)
C16	0.053 (2)	0.050 (2)	0.053 (2)	−0.0122 (16)	0.0121 (16)	0.0095 (16)
C18	0.069 (2)	0.0362 (16)	0.0493 (19)	−0.0062 (16)	0.0094 (17)	−0.0008 (14)
C2	0.057 (2)	0.050 (2)	0.056 (2)	−0.0115 (16)	0.0162 (16)	−0.0037 (16)
C24	0.053 (2)	0.065 (2)	0.070 (2)	0.0019 (18)	−0.0031 (18)	0.005 (2)
C6	0.056 (2)	0.062 (2)	0.0471 (19)	−0.0125 (17)	0.0121 (16)	−0.0123 (17)
C17	0.076 (2)	0.0365 (19)	0.065 (2)	−0.0181 (17)	0.0140 (19)	0.0019 (16)
C5	0.078 (3)	0.068 (3)	0.049 (2)	−0.011 (2)	0.0044 (19)	−0.0184 (18)
C15	0.083 (3)	0.066 (3)	0.072 (3)	−0.005 (2)	0.045 (2)	−0.003 (2)
C10	0.047 (2)	0.082 (3)	0.095 (3)	−0.001 (2)	−0.004 (2)	0.014 (3)
C22	0.0503 (19)	0.0499 (19)	0.0473 (18)	0.0109 (15)	0.0135 (15)	0.0059 (15)
C23	0.075 (3)	0.053 (2)	0.081 (3)	0.019 (2)	−0.001 (2)	0.012 (2)
C3	0.057 (2)	0.056 (2)	0.077 (3)	−0.0166 (18)	0.0062 (19)	−0.009 (2)
C8	0.078 (3)	0.084 (3)	0.061 (2)	0.033 (2)	−0.011 (2)	−0.021 (2)
C11	0.093 (3)	0.133 (5)	0.091 (3)	0.037 (3)	−0.045 (3)	−0.041 (3)
C4	0.071 (2)	0.051 (2)	0.068 (2)	−0.0086 (19)	−0.003 (2)	−0.0153 (19)
C9	0.072 (3)	0.097 (4)	0.094 (3)	0.036 (3)	−0.017 (2)	−0.019 (3)
C12	0.085 (3)	0.097 (4)	0.082 (3)	0.026 (3)	−0.026 (2)	−0.044 (3)

### Geometric parameters (Å, °)

**Table:**

Sn1—O3	2.1085 (19)	C24—H24B	0.9600
Sn1—C7	2.135 (3)	C24—H24C	0.9600
Sn1—C1	2.142 (3)	C6—C5	1.516 (4)
Sn1—O1	2.157 (2)	C6—H6A	0.9700
Sn1—N1	2.172 (2)	C6—H6B	0.9700
N1—C20	1.292 (4)	C17—H17	0.9300
N1—C21	1.481 (3)	C5—C4	1.515 (5)
O1—C25	1.289 (4)	C5—H5A	0.9700
O3—C13	1.309 (3)	C5—H5B	0.9700
O4—C14	1.360 (4)	C15—H15A	0.9600
O4—C15	1.422 (4)	C15—H15B	0.9600
O2—C25	1.217 (4)	C15—H15C	0.9600
C19—C13	1.402 (4)	C10—C9	1.472 (6)
C19—C18	1.409 (4)	C10—C11	1.487 (6)
C19—C20	1.431 (4)	C10—H10A	0.9700
C13—C14	1.424 (4)	C10—H10B	0.9700
C25—C21	1.520 (4)	C22—C23	1.521 (5)
C14—C16	1.370 (4)	C22—H22	0.9800
C7—C12	1.492 (5)	C23—H23A	0.9600
C7—C8	1.512 (5)	C23—H23B	0.9600
C7—H7	0.9800	C23—H23C	0.9600
C21—C22	1.538 (4)	C3—C4	1.516 (5)
C21—H21	0.9800	C3—H3A	0.9700
C20—H20	0.9300	C3—H3B	0.9700
C1—C6	1.530 (4)	C8—C9	1.531 (5)
C1—C2	1.530 (4)	C8—H8A	0.9700
C1—H1	0.9800	C8—H8B	0.9700
C16—C17	1.390 (5)	C11—C12	1.527 (6)
C16—H16	0.9300	C11—H11A	0.9700
C18—C17	1.359 (5)	C11—H11B	0.9700
C18—H18	0.9300	C4—H4A	0.9700
C2—C3	1.522 (5)	C4—H4B	0.9700
C2—H2A	0.9700	C9—H9A	0.9700
C2—H2B	0.9700	C9—H9B	0.9700
C24—C22	1.515 (5)	C12—H12A	0.9700
C24—H24A	0.9600	C12—H12B	0.9700
			
O3—Sn1—C7	95.63 (10)	C1—C6—H6B	109.3
O3—Sn1—C1	95.31 (9)	H6A—C6—H6B	108.0
C7—Sn1—C1	121.20 (12)	C18—C17—C16	119.9 (3)
O3—Sn1—O1	157.22 (8)	C18—C17—H17	120.0
C7—Sn1—O1	92.06 (10)	C16—C17—H17	120.0
C1—Sn1—O1	98.96 (10)	C4—C5—C6	111.8 (3)
O3—Sn1—N1	82.79 (8)	C4—C5—H5A	109.3
C7—Sn1—N1	119.27 (11)	C6—C5—H5A	109.3
C1—Sn1—N1	119.38 (10)	C4—C5—H5B	109.3
O1—Sn1—N1	74.75 (8)	C6—C5—H5B	109.3
C20—N1—C21	117.3 (2)	H5A—C5—H5B	107.9
C20—N1—Sn1	126.18 (19)	O4—C15—H15A	109.5
C21—N1—Sn1	115.66 (17)	O4—C15—H15B	109.5
C25—O1—Sn1	120.55 (19)	H15A—C15—H15B	109.5
C13—O3—Sn1	130.97 (18)	O4—C15—H15C	109.5
C14—O4—C15	117.4 (3)	H15A—C15—H15C	109.5
C13—C19—C18	120.4 (3)	H15B—C15—H15C	109.5
C13—C19—C20	123.1 (3)	C9—C10—C11	111.5 (4)
C18—C19—C20	116.6 (3)	C9—C10—H10A	109.3
O3—C13—C19	123.8 (3)	C11—C10—H10A	109.3
O3—C13—C14	118.6 (3)	C9—C10—H10B	109.3
C19—C13—C14	117.6 (3)	C11—C10—H10B	109.3
O2—C25—O1	124.7 (3)	H10A—C10—H10B	108.0
O2—C25—C21	118.6 (3)	C24—C22—C23	110.3 (3)
O1—C25—C21	116.7 (3)	C24—C22—C21	113.0 (3)
O4—C14—C16	125.1 (3)	C23—C22—C21	112.7 (3)
O4—C14—C13	114.4 (3)	C24—C22—H22	106.8
C16—C14—C13	120.5 (3)	C23—C22—H22	106.8
C12—C7—C8	110.1 (3)	C21—C22—H22	106.8
C12—C7—Sn1	114.7 (2)	C22—C23—H23A	109.5
C8—C7—Sn1	112.7 (2)	C22—C23—H23B	109.5
C12—C7—H7	106.3	H23A—C23—H23B	109.5
C8—C7—H7	106.3	C22—C23—H23C	109.5
Sn1—C7—H7	106.3	H23A—C23—H23C	109.5
N1—C21—C25	109.4 (2)	H23B—C23—H23C	109.5
N1—C21—C22	112.5 (2)	C4—C3—C2	111.4 (3)
C25—C21—C22	110.1 (3)	C4—C3—H3A	109.3
N1—C21—H21	108.2	C2—C3—H3A	109.3
C25—C21—H21	108.2	C4—C3—H3B	109.3
C22—C21—H21	108.2	C2—C3—H3B	109.3
N1—C20—C19	128.2 (3)	H3A—C3—H3B	108.0
N1—C20—H20	115.9	C7—C8—C9	111.5 (3)
C19—C20—H20	115.9	C7—C8—H8A	109.3
C6—C1—C2	110.3 (3)	C9—C8—H8A	109.3
C6—C1—Sn1	110.57 (19)	C7—C8—H8B	109.3
C2—C1—Sn1	112.0 (2)	C9—C8—H8B	109.3
C6—C1—H1	107.9	H8A—C8—H8B	108.0
C2—C1—H1	107.9	C10—C11—C12	112.0 (4)
Sn1—C1—H1	107.9	C10—C11—H11A	109.2
C14—C16—C17	121.0 (3)	C12—C11—H11A	109.2
C14—C16—H16	119.5	C10—C11—H11B	109.2
C17—C16—H16	119.5	C12—C11—H11B	109.2
C17—C18—C19	120.6 (3)	H11A—C11—H11B	107.9
C17—C18—H18	119.7	C5—C4—C3	110.5 (3)
C19—C18—H18	119.7	C5—C4—H4A	109.5
C3—C2—C1	110.6 (3)	C3—C4—H4A	109.5
C3—C2—H2A	109.5	C5—C4—H4B	109.5
C1—C2—H2A	109.5	C3—C4—H4B	109.5
C3—C2—H2B	109.5	H4A—C4—H4B	108.1
C1—C2—H2B	109.5	C10—C9—C8	111.6 (4)
H2A—C2—H2B	108.1	C10—C9—H9A	109.3
C22—C24—H24A	109.5	C8—C9—H9A	109.3
C22—C24—H24B	109.5	C10—C9—H9B	109.3
H24A—C24—H24B	109.5	C8—C9—H9B	109.3
C22—C24—H24C	109.5	H9A—C9—H9B	108.0
H24A—C24—H24C	109.5	C7—C12—C11	112.3 (4)
H24B—C24—H24C	109.5	C7—C12—H12A	109.1
C5—C6—C1	111.6 (3)	C11—C12—H12A	109.1
C5—C6—H6A	109.3	C7—C12—H12B	109.1
C1—C6—H6A	109.3	C11—C12—H12B	109.1
C5—C6—H6B	109.3	H12A—C12—H12B	107.9
			
O3—Sn1—N1—C20	−20.0 (2)	O2—C25—C21—N1	164.9 (3)
C7—Sn1—N1—C20	72.5 (3)	O1—C25—C21—N1	−16.1 (4)
C1—Sn1—N1—C20	−112.0 (3)	O2—C25—C21—C22	−70.9 (4)
O1—Sn1—N1—C20	156.2 (3)	O1—C25—C21—C22	108.0 (3)
O3—Sn1—N1—C21	171.3 (2)	C21—N1—C20—C19	−177.8 (3)
C7—Sn1—N1—C21	−96.3 (2)	Sn1—N1—C20—C19	13.6 (5)
C1—Sn1—N1—C21	79.2 (2)	C13—C19—C20—N1	0.6 (5)
O1—Sn1—N1—C21	−12.62 (19)	C18—C19—C20—N1	−179.7 (3)
O3—Sn1—O1—C25	13.5 (4)	O3—Sn1—C1—C6	46.7 (2)
C7—Sn1—O1—C25	123.3 (2)	C7—Sn1—C1—C6	−53.2 (3)
C1—Sn1—O1—C25	−114.6 (2)	O1—Sn1—C1—C6	−151.1 (2)
N1—Sn1—O1—C25	3.5 (2)	N1—Sn1—C1—C6	131.4 (2)
C7—Sn1—O3—C13	−94.7 (3)	O3—Sn1—C1—C2	170.2 (2)
C1—Sn1—O3—C13	143.1 (3)	C7—Sn1—C1—C2	70.4 (2)
O1—Sn1—O3—C13	14.4 (4)	O1—Sn1—C1—C2	−27.6 (2)
N1—Sn1—O3—C13	24.1 (3)	N1—Sn1—C1—C2	−105.1 (2)
Sn1—O3—C13—C19	−20.1 (4)	O4—C14—C16—C17	−179.5 (3)
Sn1—O3—C13—C14	161.1 (2)	C13—C14—C16—C17	0.0 (5)
C18—C19—C13—O3	−177.8 (3)	C13—C19—C18—C17	−0.4 (5)
C20—C19—C13—O3	2.0 (5)	C20—C19—C18—C17	179.8 (3)
C18—C19—C13—C14	1.0 (4)	C6—C1—C2—C3	−55.8 (4)
C20—C19—C13—C14	−179.3 (3)	Sn1—C1—C2—C3	−179.4 (2)
Sn1—O1—C25—O2	−175.0 (3)	C2—C1—C6—C5	54.9 (4)
Sn1—O1—C25—C21	6.1 (4)	Sn1—C1—C6—C5	179.3 (3)
C15—O4—C14—C16	−2.0 (5)	C19—C18—C17—C16	−0.4 (6)
C15—O4—C14—C13	178.5 (3)	C14—C16—C17—C18	0.6 (6)
O3—C13—C14—O4	−2.4 (4)	C1—C6—C5—C4	−55.1 (4)
C19—C13—C14—O4	178.8 (3)	N1—C21—C22—C24	58.4 (4)
O3—C13—C14—C16	178.0 (3)	C25—C21—C22—C24	−63.9 (3)
C19—C13—C14—C16	−0.8 (4)	N1—C21—C22—C23	−67.5 (4)
O3—Sn1—C7—C12	80.8 (3)	C25—C21—C22—C23	170.2 (3)
C1—Sn1—C7—C12	−179.5 (3)	C1—C2—C3—C4	57.4 (4)
O1—Sn1—C7—C12	−77.7 (3)	C12—C7—C8—C9	54.6 (5)
N1—Sn1—C7—C12	−4.0 (3)	Sn1—C7—C8—C9	−176.1 (3)
O3—Sn1—C7—C8	−46.1 (3)	C9—C10—C11—C12	−54.3 (6)
C1—Sn1—C7—C8	53.6 (3)	C6—C5—C4—C3	55.4 (4)
O1—Sn1—C7—C8	155.4 (3)	C2—C3—C4—C5	−56.7 (4)
N1—Sn1—C7—C8	−131.0 (3)	C11—C10—C9—C8	55.3 (6)
C20—N1—C21—C25	−151.0 (3)	C7—C8—C9—C10	−56.0 (5)
Sn1—N1—C21—C25	18.8 (3)	C8—C7—C12—C11	−53.9 (5)
C20—N1—C21—C22	86.3 (3)	Sn1—C7—C12—C11	177.8 (4)
Sn1—N1—C21—C22	−103.9 (2)	C10—C11—C12—C7	54.3 (7)

## References

[bb1] Basu Baul, T. S., Masharing, C., Ruisi, G., Jirasko, R., Holcapek, M., De Vos, D., Wolstenholme, D. & Linden, A. (2007). *J. Organomet. Chem.* **692**, 4849–4862.

[bb2] Beltran, H. I., Zamudio-Rivera, L. S., Mancilla, T., Santillan, R. & Farfan, N. (2003). *Chem. Eur. J.* **9**, 2291–2306. 10.1002/chem.200204260 12772304

[bb3] Bruker (2002). *SADABS*, *SAINT* and *SMART* Bruker AXS Inc., Madison, Wisconsin, USA.

[bb4] Dakternieks, D., Basu Baul, T. S., Dutta, S. & Tiekink, E. R. T. (1998). *Organometallics*, **17**, 3058–3062.

[bb5] Farrugia, L. J. (1997). *J. Appl. Cryst.* **30**, 565.

[bb6] Li, J.-P. & Tian, L.-J. (2008). *Acta Cryst.* E**64**, m98. 10.1107/S1600536807064471 PMC2914970 21200663

[bb7] Sheldrick, G. M. (2008). *Acta Cryst.* A**64**, 112–122. 10.1107/S0108767307043930 18156677

[bb8] Tian, L., Liu, X., Shang, Z., Li, D. & Yu, Q. (2004). *Appl. Organomet. Chem.* **18**, 483–484.

[bb9] Tian, L., Qian, B., Sun, Y., Zheng, X., Yang, M., Li, H. & Liu, X. (2005). *Appl. Organomet. Chem.* **19**, 980–987.

[bb10] Tian, L., Shang, Z., Zheng, X., Sun, Y., You, Y., Qian, B. & Liu, X. (2006). *Appl. Organomet. Chem.* **20**, 74–80.

[bb11] Tian, L., Sun, Y., Zheng, X., Liu, X., You, Y., Liu, X. & Qian, B. (2007). *Chin. J. Chem.* **25**, 312–318.

[bb12] Tian, L., Yang, H., Zheng, X., Ni, Z., Yan, D., Tu, L. & Jiang, J. (2009). *Appl. Organomet. Chem.* **22**, 24–31.

